# Ultrasonic Plasma Engineering Toward Facile Synthesis of Single-Atom M-N_4_/N-Doped Carbon (M = Fe, Co) as Superior Oxygen Electrocatalyst in Rechargeable Zinc–Air Batteries

**DOI:** 10.1007/s40820-020-00581-4

**Published:** 2021-01-21

**Authors:** Kai Chen, Seonghee Kim, Minyeong Je, Heechae Choi, Zhicong Shi, Nikola Vladimir, Kwang Ho Kim, Oi Lun Li

**Affiliations:** 1 Department of Materials Science and Engineering, Pusan National University, 30 Jangjeon-dong, Geumjeong-gu, Busan, 609-735 Republic of Korea; 2 Theoretical Materials and Chemistry Group, Institute of Inorganic Chemistry, University of Cologne, Greinstr. 6, 50939 Cologne, Germany; 3 School of Materials and Energy, Guangdong University of Technology, Guangzhou, 510006 People’s Republic of China; 4 Faculty of Mechanical Engineering and Naval Architecture, University of Zagreb, Ivana Lucica 5, 10002 Zagreb, Croatia; 5 Global Frontier R&D Center for Hybrid Interface Materials, 30 Jangjeon-dong, Geumjeong-gu, Busan, 46241 Republic of Korea

**Keywords:** Single-atom-doped M-N_4_/NC catalyst, Plasma engineering, ORR/OER bifunctional activity, DFT calculation, Rechargeable Zn–air battery

## Abstract

As bifunctional oxygen evolution/reduction electrocatalysts, transition-metal-based single-atom-doped nitrogen–carbon (NC) matrices are promising successors of the corresponding noble-metal-based catalysts, offering the advantages of ultrahigh atom utilization efficiency and surface active energy. However, the fabrication of such matrices (e.g., well-dispersed single-atom-doped M-N_4_/NCs) often requires numerous steps and tedious processes. Herein, ultrasonic plasma engineering allows direct carbonization in a precursor solution containing metal phthalocyanine and aniline. When combining with the dispersion effect of ultrasonic waves, we successfully fabricated uniform single-atom M-N_4_ (M = Fe, Co) carbon catalysts with a production rate as high as 10 mg min^−1^. The Co-N_4_/NC presented a bifunctional potential drop of Δ*E* = 0.79 V, outperforming the benchmark Pt/C-Ru/C catalyst (Δ*E* = 0.88 V) at the same catalyst loading. Theoretical calculations revealed that Co-N_4_ was the major active site with superior O_2_ adsorption–desorption mechanisms. In a practical Zn–air battery test, the air electrode coated with Co-N_4_/NC exhibited a specific capacity (762.8 mAh g^−1^) and power density (101.62 mW cm^−2^), exceeding those of Pt/C-Ru/C (700.8 mAh g^−1^ and 89.16 mW cm^−2^, respectively) at the same catalyst loading. Moreover, for Co-N_4_/NC, the potential difference increased from 1.16 to 1.47 V after 100 charge–discharge cycles. The proposed innovative and scalable strategy was concluded to be well suited for the fabrication of single-atom-doped carbons as promising bifunctional oxygen evolution/reduction electrocatalysts for metal–air batteries.

**Supplementary Information:**

The online version contains supplementary material available at 10.1007/s40820-020-00581-4

## Highlights

- Single-atom M-N_4_/N-doped carbons (M = Fe, Co) prepared as OER/ORR catalysts.
- Ultrasonication-assisted plasma engineering used for catalyst synthesis.
- Co-N_4_/NC outperformed benchmark commercial catalysts in practical Zn–air battery test.
- DFT calculations provided insights into the origin of superior ORR/OER performance.

**Supplementary Information:**

The online version contains supplementary material available at 10.1007/s40820-020-00581-4

## Graphical abstract

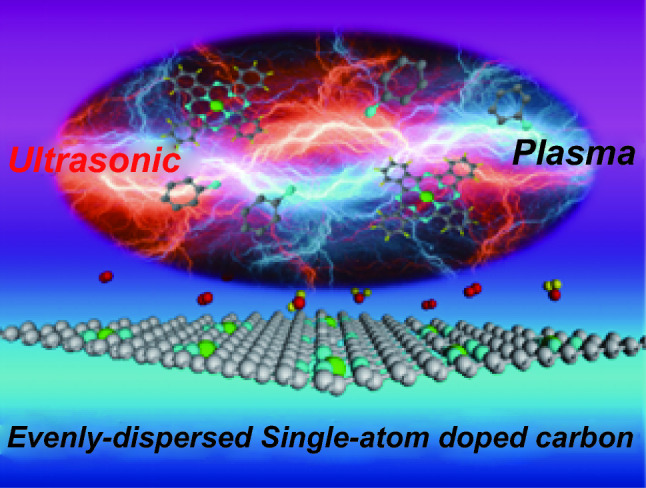

## Introduction

The growing demand for green and renewable energy has inspired the vigorous development of power conversion and storage devices (e.g., fuel cells as well as metal–air and secondary batteries) that reveal high power densities and are cheap, safe to handle, and eco-friendly [1–5]. Among the numerous energy conversion devices, rechargeable zinc–air batteries (ZABs) feature a decent theoretical density (1084 Wh kg^−1^) and high safety; thus, they are potentially suited for application in electric vehicles and portable electronic devices [5, 6]. However, the widespread commercialization of these batteries is hindered by their large overpotentials for the oxygen reduction reaction (ORR) and the oxygen evolution reaction (OER) [7]. Given that noble-metal-based ORR/OER electrocatalysts (such as Pt and Ru/Ir) suffer from high cost and poor stability during repeated charge–discharge [8], it is essential to develop new bifunctional ORR/OER catalysts with high activity and cycling stability and containing earth-abundant elements as replacements of noble-metal-based catalysts in rechargeable ZABs [9].

Transition-metal-based single-atom-doped nitrogen–carbon electrocatalysts (M-N_4_/NCs) are promising successors of noble-metal-based nanocatalysts, exhibiting excellent ORR/OER activity due to their ultrahigh atom utilization efficiency and surface active energy [1]. The superior catalytic activity of M-N_4_/NCs mainly originates from the specific coordination between transition metal atoms and nitrogen, which provides a highly efficient means of modifying local electronic states [10, 11]. For instance, Han et al. [1] presented a single-atom Fe–N_*x*_–C ORR electrocatalyst with a high positive half-wave potential and open-circuit voltage, while Zhu et al. [12] prepared hierarchically porous M–N_*x*_–C (M = Co, Fe) single-atom electrocatalysts with superior ORR activity as a result of the rise active sites. Thus, the introduction of transition metals as monoatom active sites into N-doped carbon (NC) matrices is a suitable way of improving ZAB performance. Despite these achievements, the fabrication of well-dispersed M-N_4_/NCs remains challenging. Notably, control of the interfacial contact between the metal atoms and the charcoal support as well as that of metal atom dispersion are crucial for ORR/OER activity enhancement. The currently known M-N_4_/NC catalysts are largely synthesized by pyrolysis of transition-metal-based metal–organic frameworks (MOFs) [13–17] and metal phthalocyanines [18, 19] or by the chemical polymerization of metal-salt-impregnated organic precursors, such as urea, melamine, or aniline, followed by pyrolysis with a conductive carbon matrix at 700–1000 °C [20–24]. Moreover, rigid structures like zeolite imidazole frameworks [25, 26] or functionalized carbons with oxygenated groups should be used as supports to immobilize metal ions and thus prevent the aggregation of metal species during thermal treatment [27]. Although these methods allow one to obtain carbon matrices with various atomically dispersed metal centers, they require numerous steps and tedious processes to synthesize appropriate precursors, and the corresponding carbonization ratios and production rates remain low [28]. In addition, the decrease in particle size to the single-atom level often increases surface free energy and thus facilitates the aggregation of metal active sites to form larger clusters or nanoparticles at high pyrolysis temperatures. Consequently, one should establish a facile and scalable strategy of incorporating single metal atoms during carbon synthesis and aim to reduce the temperature of thermal treatment to prevent the aggregation of these atoms into clusters.

Unlike conventional syntheses of single-metal-atom-doped carbon, which require high annealing temperatures for complete carbonization, plasma engineering allows direct carbonization in a precursor solution in an organic solvent (e.g., benzene) [29] or in a solution containing nitrogen and carbon (e.g., pyridine, phenylamine) [30, 31] at ambient temperature and pressure. Specifically, the generation of NC can be achieved via rapid C–N and/or C–C integration during plasma discharge and the related chemical reactions [31]. For instance, B–N double-site-doped carbon was prepared by supplementing the precursors with boric acid and/or phenylboronic acid [31, 32]. The mechanisms of the in situ syntheses of heteroatom-doped carbons via plasma engineering have been thoroughly reviewed by Li et al. [33]. More importantly, Morishita et al. reported that the rate of carbon production from benzene derivatives (e.g., benzene and aniline) via plasma synthesis (1–10 mg min^−1^) substantially exceeds that of pyrolytic methods [34]. Thus, it was hypothesized that plasma engineering can be used to effectively prepare single-atom-doped catalysts from aniline and metal phthalocyanines. During plasma engineering, direct carbonization proceeds by the combination of CN and C_2_ radicals with the solution precursor at the gas/liquid interface. Since the metal phthalocyanine has high chemical stability and is only present in small amount in the solution phase, the molecular structure is expected to maintain its planar metal-N_4_ coordination during the fast formation of carbon materials. Also, the solubility of metal phthalocyanines is very low in pure aniline, which might result in easy agglomeration. An ultrasonic homogenizer uses the dispersion effect of ultrasonic waves in the liquid to cause cavitation of the liquid, which is useful in mixing, emulsifying, dispersing, and deagglomeration [35]. To the best of our knowledge, this is the first study to combine plasma engineering with ultrasonication-assisted homogenization to prevent agglomeration during the synthesis and to successfully dope molecular M-N_4_ atomically within the NC matrix with a high production rate of ~ 10 mg min^−1^.

Among the screened metal phthalocyanines, M-N_4_ structures featuring Fe and Co as central metal atoms exhibited the best ORR/OER catalytic activity in an alkaline electrolyte [36]. Wang et al. [37] applied iron phthalocyanine (Fe-Pc) as an analog to FeN_4_ and explored the ORR activity by means of comprehensive density functional theory (DFT) computations. It was found that O_2_ could readily bind to the Fe center with charge transfer from Fe to O_2_, and O_2_* reduction was identified as the rate-determining step. Moreover, Peng et al. [38] indicated that the number of states around the Fermi level was significantly higher for the FeN_4_ moiety with dominant contributions from the Fe 3d orbital based on DFT density of states plots. This indicates that the Fe center is favorable for the adsorption of O_2_ and may donate electrons to reduce O_2_, which results in superior ORR catalytic activity. In contrast, the contribution of Co-N_4_ seems to be more significant in OER process. Fei et al. investigated the MN_4_C_4_ (M = Fe, Co, and Ni) moieties in a graphene matrix by DFT calculations and experimental studies [39]. In the theoretical calculations, the free energy of the rate-determining step (RDS) for Fe–NHGF is the oxidation of O* to OOH* with limiting barrier energy as large as 0.97 eV. In the case of Co–NHGF, the RDS is the oxidation of OH* to O* with much lower limiting barrier of 0.52 eV. The experimental results also agreed with the DFT calculation and supported the advanced OER catalytic activity in Co moieties.

Herein, single-atom Fe-N_4_/NC and Co-N_4_/NC catalysts were prepared via ultrasonication-assisted plasma engineering. High-purity aniline was used as a solvent and NC matrix precursor, while the metal phthalocyanine (M = Fe, Co) acted as a coupling agent and precursor to form the M-N_4_ matrix. As the thermal decomposition of these phthalocyanines normally occurs at 570–750 °C [40], a mild annealing temperature of 550 °C was chosen for the single-atom catalyst synthesis to enhance the graphitization of NC and preserve the M-N_4_ molecular structure. The very low solubility of metal phthalocyanines in pure aniline results in easy agglomeration under typical plasma synthesis conditions, if there are no external forces to keep these large molecules separated. Thus, an ultrasonic homogenizer was additionally employed to ensure the atomic-level dispersion of M-N_4_ within the nitrogen–carbon matrix during plasma engineering. To verify the importance of homogenization, Co-N_4_/NC was synthesized in the absence of the above homogenizer under otherwise identical conditions, and Co nanoparticles were detected in the resulting NC matrix (Fig. S1a–e). The corresponding X-ray diffraction (XRD) pattern featured peaks at 2θ = 44°, 52°, and 76° that were ascribed to the [110], [200], and [220] planes of Co metal, respectively (Fig. S1f). Notably, our novel strategy allowed the facile synthesis of atomically dispersed M-N_4_/NC (M = Fe, Co) electrocatalysts with ORR/OER activities and stabilities superior to those of commercial noble-metal-based catalysts. Specifically, Co-N_4_/NC exhibited a bifunctional potential drop (Δ*E* = *E*_*j*=10_ − *E*_1/2_ = 0.79 V) superior to that of the benchmark Pt/C-Ru/C (Δ*E*_Pt/C-Ru/C_ = 0.88 V) at the same loading. Free energy changes determined by density functional theory (DFT) calculations suggested that the O_2_ adsorption–desorption performance of Co-N_4_/NC is superior to that of the well-studied Fe-N_4_/NC, in line with experimental results. Finally, in rechargeable ZABs, the Co-N_4_/NC-based air electrode achieved higher power density, higher specific capability, and better durability than that constructed using a commercial Pt/C-Ru/C catalyst.

## Experimental

### Synthesis of M-N_4_/N-Doped Carbons

The metal (Fe, Co) phthalocyanine (~ 95%, Sigma-Aldrich, Korea) was dissolved in aniline (100 mL; > 99%, Junsei Chemical Co., Ltd., Japan) for 1 h upon magnetic stirring to achieve a concentration of 1 mM. Then, two high-purity graphite electrodes were discharged in this solution at a voltage of 1.2 kV, a frequency of 25 kHz, and a pulse width of 0.9 μs for 20 min (Pulse Modulator, MPP04-A4-200. Japan), with the assistance of an ultrasonic homogenizer (Korea Process Technology Co., Ltd., KSC-80, 25 kHz) [31]. The liquid phase was passed through a 55-mm-diameter polytetrafluoroethylene filter and evaporated at 80 °C for 10 h. The resulting product was heated at 550 °C for 2 h in N_2_ to enhance conductance and denoted as Fe-N_4_/NC and Co-N_4_/NC.

### Electrocatalyst Characterization

Morphologies and elemental distributions were probed by field-emission scanning transmission electron microscopy (FE-STEM; HD-2300A, Hitachi, Japan) at an operating voltage of 200 kV. Individual elements were identified by electron energy loss spectroscopy (EELS; JEM-ARM200F, JEOL, Japan). Crystalline phases were identified by XRD analysis (Ultima IV, Rigaku, Japan). Nanocomposite graphitization degree, irregularities, and imperfections were characterized by Raman spectroscopy (VERTEX 80v, Bruker, Korea). Surface chemical states were identified by X-ray photoelectron spectroscopy (XPS; Kratos Analytical Ltd., Axis Supra, UK). The absolute metal contents of carbon catalysts were probed by inductively coupled plasma optical emission spectrometry (Optima 8300, Perkin Elmer, USA). Fe K-edge- and Co K-edge-extended X-ray absorption fine structure (EXAFS) spectra were recorded according to fluorescent patterns using the TPS 44A1 beamline (situated in the National Synchrotron Radiation Research Center (NSRRC) of Taiwan, China) at an energy of 3 GeV and an average current of 250 mA [41]. The radioactive ray was monochromatized by a monochromator with Si (111) bicrystal. X-ray absorption near-edge structure (XANES) and EXAFS data reduction and analysis were performed using Athena software [41].

### Electrochemical Measurements

The electrochemical characteristics of ORR/OER catalysts were investigated using a benchmark three-electrode system and an electrochemical workstation (Biologic, VSP, France) [31]. All measurements were conducted in 0.1 M KOH (specification of analysis, Samchun Co., Ltd.) solution at ambient temperature and pressure. Typically, M-N_4_/NC (4 mg) was dispersed into the solution of deionized water (480 μL), ethanol (480 μL, 99.9%, Duksan Co., Ltd.), and Nafion^®^117 (40 μL, 5 wt %, Aldrich Co., Ltd.) by 30-min ultrasonication to produce a well-distributed catalyst ink. The ink was drop-cast onto a well-polished glassy carbon disk electrode (*R*_diameter_ = 4 mm) and dried under ambient conditions to afford a loading of 800 μg/cm^2^. A Pt wire was used as a counter electrode, while Hg/HgO in 1 M NaOH was regarded as a reference electrode. Every electric potential was calibrated using the reversible hydrogen electrode (RHE) conversion formula, *E*_RHE_ (V) = *E*_Hg/HgO(1MNaOH)_ + 0.198 + 0.059pH. For ORR analysis, polarization curves were constructed using linear sweep voltammetry (LSV) measurements in O_2_-saturated 0.1 M KOH at a scanning speed (5 mV s^−1^) and a rotation rate (1600 rpm) within a voltage region (0.2–1.2 V vs. RHE). Chronoamperometry (CA) measurement of Co-N_4_/NC was performed at 0.6 V versus RHE for 30,000 s to examine cycling stability and catalyst durability. Also, chronopotentiometry (CP) response at a certain current density (–4 mA cm^−2^) for the Co-N_4_/NC was measured to verify the durability and stability. Cyclic voltammetry (CV) measurements were carried out at 100 mV s^−1^ in the voltage range 0.4–1.0 V versus RHE for 3000 cycles. Additionally, the average electron transfer number (*n*) and the yield of H_2_O_2_ were measured based on rotating ring–disk electrodes (RRDE) analysis. It is expressed by the following equation:

1 $$n = 4 \times I_{\text{d}} /\left( {I_{\text{d}} + I_{\text{r}} /N} \right)$$

2 $${\text{H}}_{ 2} {\text{O}}_{ 2} \,\left( \% \right) = 200 \, \times I_{\text{r}} \times N^{ - 1} /\left( {I_{\text{d}} + I_{\text{r}} /N} \right)$$

where *I*_d_, *I*_r_, and *N* denote the disk current, ring current, and current collection efficiency, respectively.

For OER analysis, LSV measurements were conducted in O_2_-saturated 0.1 M KOH at a scan rate of 5 mV s^−1^ and a rotation rate of 1600 rpm within the potential range 1–1.8 V versus RHE. CA measurements were performed at 1.6 V versus RHE for 5000 s to investigate catalyst cycling stability, while CV measurements were performed at 100 mV s^−1^ for 3000 cycles in the potential range 1–1.6 V versus RHE. Electrochemical impedance spectra were recorded in a frequency range from 100 kHz to 100 mHz at 0.9 V versus RHE. For comparison, 20 wt % Pt/C (product code: 591278-1, Fuel Cell Store, USA) and 5 wt % Ru/C (product code: 1002653245, Aldrich Co., Ltd. USA) were used as benchmark ORR and OER catalysts, respectively.

### DFT Calculations

DFT calculations were performed using the Vienna ab initio software package [42, 43]. Interactions between valence and core electrons were modeled using the projector-augmented wave method [44]. The plane-basis wave expansion was analyzed using the generalized gradient approximation with the Perdew–Burke–Ernzerhof functional [45]. The cutoff energy was 400 eV. The Brillouin area was divided by the sampled grids of 2 × 2 × 1 gamma-centered *k*-points in both vertical and lateral graphene supercells with embedded M-N_4_ (M = Fe, Co) structures [46]. A smearing width of 0.1 eV was used in the Methfessel–Paxton smearing scheme [42]. In the self-consistent calculation, the energy convergence domain was set to 10^−5^ eV, and the geometric configuration was completely relaxed until Hellman–Feynman forces reached 0.02 eV Å^−1^. The binding energies of OH, O, OOH, and O_2_ were calculated to determine the OER- and ORR-induced energy changes of M-N_4_ (M = Fe, Co). The Gibbs free energy (Δ*G*) change of the response was calculated as:

3 $$\Delta G = \Delta E + \Delta {\text{ZPE}}{-}T\Delta S + \Delta G_{\text{U}} + 0.059\,{\text{pH}}$$

where Δ*E* is the calculated total energy difference, and ΔZPE, *T*Δ*S*, and Δ*G*_U_ are the zero-point energy correction, entropy contribution, and free energy term related to the applied electrode potential *U*, respectively.

### Rechargeable ZAB Testing

Testing was performed with the as-prepared electrocatalysts loaded on the gas diffusion layer of the air electrode of an in-house-made ZAB at room temperature. The catalyst ink was prepared as described above and drop-cast onto carbon paper at a mass loading of 1 mg cm^−2^. The air cathode was coated with a 1:1 (w/w) mixture of 20 wt % Pt/C and 5 wt % Ru/C at the same total catalyst loading. Zn foil was used as the metal anode, and a solution containing 6 M KOH (Guaranteed Reagent, Junsei Co., Ltd.) and 0.2 M zinc acetate (Sigma-Aldrich Co., Ltd.) was used as the electrolyte. Polarization curves were constructed by performing LSV measurements at a sweep rate of 10 mV s^−1^ using an electrochemical workstation (Biologic, VSP, France). Specific capacity and energy density were calculated as [47]

4 $${\text{Energy}}\,{\text{density}}\,({\text{mW}}\,{\text{cm}}^{ - 2} ) = {\text{IV}}\Delta t/\omega_{\text{Zn}}$$

5 $${\text{Specific}}\,{\text{capacity}}\,({\text{mA}}\,{\text{h}}\,{\text{g}}^{ - 1} ) = I\Delta t/\omega_{\text{Zn}}$$

where *I*, *V*, Δ*t*, and *ω*_Zn_ represent the current, average discharge voltage, testing time, and amount of consumed Zn, respectively.

Galvanostatic charge–discharge behavior was characterized using the same system. In the cycling test, 100 charge–discharge (5 min + 5 min) cycles were performed at a current density of 10 mA cm^−2^.

## Results and Discussion

### Catalyst Fabrication and Characterization

**Fig. 1:**
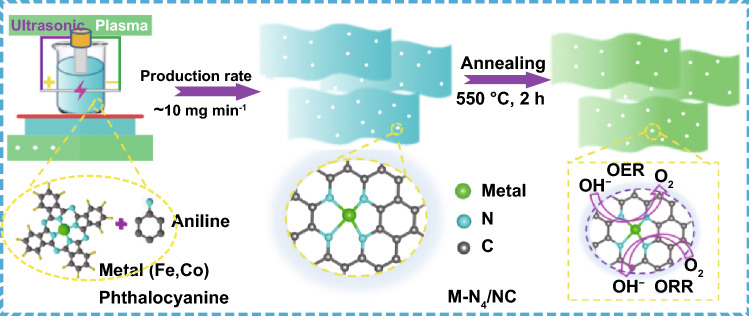
Schematic of ultrasonic plasma engineering fabrication of M-N_4_/NC (M = Fe, Co)

**Fig. 2:**
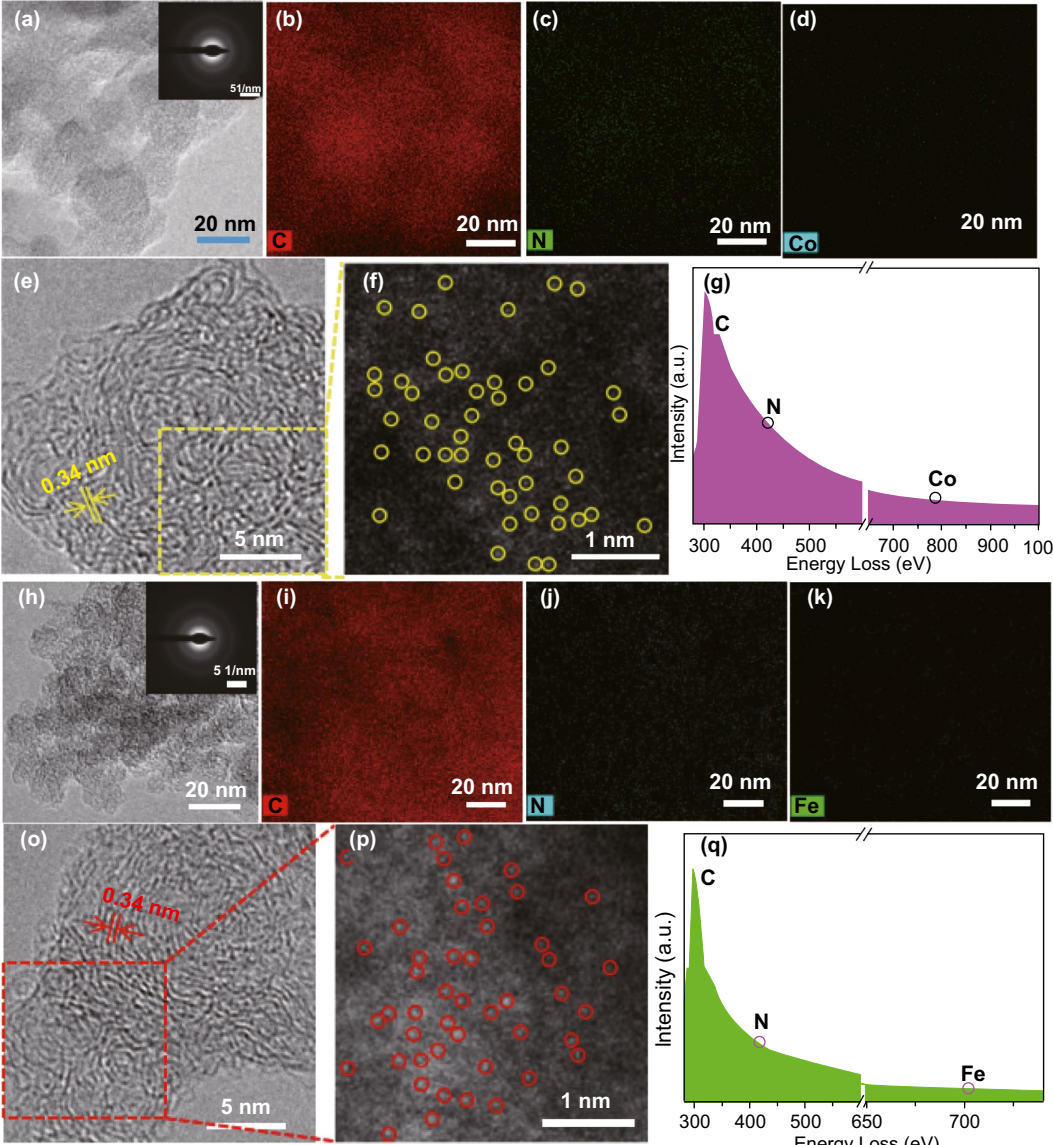
**a**–**d** TEM image of Co-N_4_/NC (inset: SAED pattern) and elemental mapping of Co, N, and C. **e** HR-TEM (bright field). **f** Aberration-corrected HAADF-TEM image. Enlarged pattern of Co-N_4_/NC and partial single Co atoms (yellow). **g** EELS atomic spectra of Co, N, and C elements corresponding to **f**. **h**–**k** TEM image of Fe-N_4_/NC (inset: SAED pattern) and elemental mapping of Fe, N, and C. **o** HR-TEM (bright field). **p** Aberration-corrected HAADF-TEM image of Fe-N_4_/NC and partial single Fe atoms (red). **q** EELS atomic spectra of Fe, N, and C elements corresponding to **p**

Figure 1 presents the synthesis of M-N_4_/NC (M = Fe, Co) electrocatalysts via ultrasonication-assisted plasma engineering. The prepared M-N_4_/NCs were converted into isolated single-atom M-N_4_/NCs by 2-h annealing in N_2_ at 550 °C. In addition, NC was synthesized from pure aniline as a control. The production rates of M-N_4_/NC and NC were close to 10 mg min^−1^, which agreed with values reported for similar processes [33, 34]. Transmission electron microscopy (TEM) imaging and elemental mappings (Fig. 2a–d and h–k, respectively) showed that Co-N_4_/NC and Fe-N_4_/NC contained the respective metal, N, and C. The selected area electron diffraction (SAED) patterns (insets in Fig. 2a, h) had ringlike features indicative of poor crystallinity and the absence of metal nanocrystals. High-resolution transmission electron microscopy (HR-TEM) bright-field imaging demonstrated the presence of several graphitic carbon layers with a lattice spacing of ~ 0.34 nm directed toward the (002) plane of Co-N_4_/NC and Fe-N_4_/NC (Fig. 2e, o, respectively). Aberration-corrected high-angle annular dark-field scanning transmission electron microscopy (HAADF-STEM) images of Co-N_4_/NC and Fe-N_4_/NC revealed the presence of isolated single-atom Co and Fe sites (Fig. 2f, p, respectively). The atomic-level distributions of Co and Fe were further characterized by EELS. When the electron beam was directed at various positions (yellow regions in Fig. 2f and red regions in Fig. 2p), the obtained EELS spectra showed distinct small peaks of atomic Co and Fe (Fig. 2g, q, respectively). The metal contents of Co-N_4_/NC and Fe-N_4_/NC were determined by inductively coupled plasma atomic emission spectrometry as 0.26 and 0.29 wt %, respectively. The above findings support the successful fabrication of dispersed single-atom Co-N_4_/NC and Fe-N_4_/NC electrocatalysts.

The XRD patterns of as-prepared M-N_4_/NCs and pristine NC (Fig. S3) featured similar broad peaks at 23° and 43°, which were indexed to the (002) and (101) planes of graphitic carbon, respectively. Notably, no peaks attributable to crystalline metal nanoparticles were detected. Furthermore, the peak of the (002) plane of graphitic carbon in M-N_4_/NCs was slightly shifted to the right compared to that of NC, which indicated that the introduction of single-atom M-N_4_ units increased the average size of defects in the carbon matrix. The Raman spectra of M-N_4_/NCs and NC (Fig. S4) featured two major characteristic peaks, viz. the D-band (1344 cm^−1^) and the G-band (1587 cm^−1^), with the *I*_D_/*I*_G_ intensity ratios of Co-N_4_/NC and Fe-N_4_/NC determined to be 1.00 and 1.01, respectively. In contrast, a smaller value of 0.91 was obtained for NC. As the incorporation of single-atom M-N_4_ units created larger defects within the NC matrix, it is reasonable that Co-N_4_/NC and Fe-N_4_/NC presented a higher *I*_D_/*I*_G_ ratio than NC. The N_2_ adsorption–desorption isotherms of M-N_4_/NCs and NC (Fig. S5) could be classified as type IV, indicating a mesoporous structure. Moreover, the narrow hysteresis loop in the region of *p*/*p*_0_ > 0.9 suggested the existence of larger mesopores ascribed to the interior of graphene nanospheres. The Brunauer–Emmett–Teller surface areas of Co-N_4_/NC and Fe-N_4_/NC were estimated as 257.91 and 275.68 m^2^ g^−1^, respectively (Fig. S6 and Table S2). The relatively large surface area of M-N_4_/NCs can dramatically diminish the proton spread path and accelerate ion diffusion between the electrode and the electrolyte [48]. Combined with morphological and structural analyses, plasma engineering offers a facile route to NC matrices containing well-dispersed single metal atoms.

**Fig. 3:**
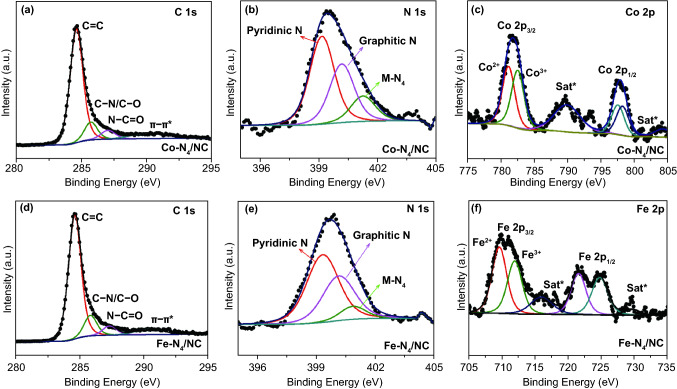
XPS narrow scan of **a** C 1s, **b** N 1s, and **c** Co 2p for Co-N_4_/NC. XPS narrow scan of **d** C 1s, **e** N 1s, and **f** Fe 2p for Fe-N_4_/NC

Survey X-ray photoelectron spectra (Fig. S7) indicated the coexistence of metals (Fe, Co) and N in the graphitic carbon networks of Co-N_4_/NC and Fe-N_4_/NC. Figure 3 provides further insights into the chemical states of each element, presenting the C, N, and Co/Fe narrow-scan spectra of M-N_4_/NCs. The high-resolution C 1s spectra of Co-N_4_/NC and Fe-N_4_/NC (Fig. 3a, d), respectively) featured three main peaks attributed to *sp*^2^ C = C (284.6 eV), C–N/C–O (285.1 eV), and N–C = O (286.8 eV). The high-resolution N 1s spectra of Co-N_4_/NC and Fe-N_4_/NC (Fig. 3b, e, respectively) showed three major peaks at 398.7, 400.5, and 401.0 eV, which corresponded to pyridinic, graphitic, and metal-bound nitrogen, respectively. In particular, the sharp peak of metal-bound nitrogen confirmed the existence of single-atom active sites, which are expected to contribute to enhanced ORR and OER activities [49]. The high-resolution Co 2p spectrum of Co-N_4_/NC (Fig. 3c) showed spin–orbit doublets at 781.6 eV (Co 2p_3/2_) and 796.3 eV (2p_1/2_), as well as two satellite peaks at 788.4 and 802.5 eV. The peak spacing of 14.7 eV between the two peaks of Co 2p_3/2_ and Co 2p_1/2_ indicated the presence of both Co^2+^ and Co^3+^ [50]. Based on the fitting of the Co 2p_3/2_ peak, the surface atomic ratio of Co^2+^/Co^3+^ was calculated as 1.54. Similarly, the high-resolution Fe 2p spectrum (Fig. 3d) featured two spin–orbit doublets at 711.3 eV (Fe 2p_3/2_, split into two small peaks of Fe^2+^ and Fe^3+^) and 722.8 eV (Fe 2p_1/2_). The Fe^2+^/Fe^3+^ surface atomic ratio was calculated as 1.26 from the results of Fe 2p_3/2_ peak fitting. The above findings confirmed the presence of M–N bonds in M-N_4_/NCs [51].

**Fig. 4:**
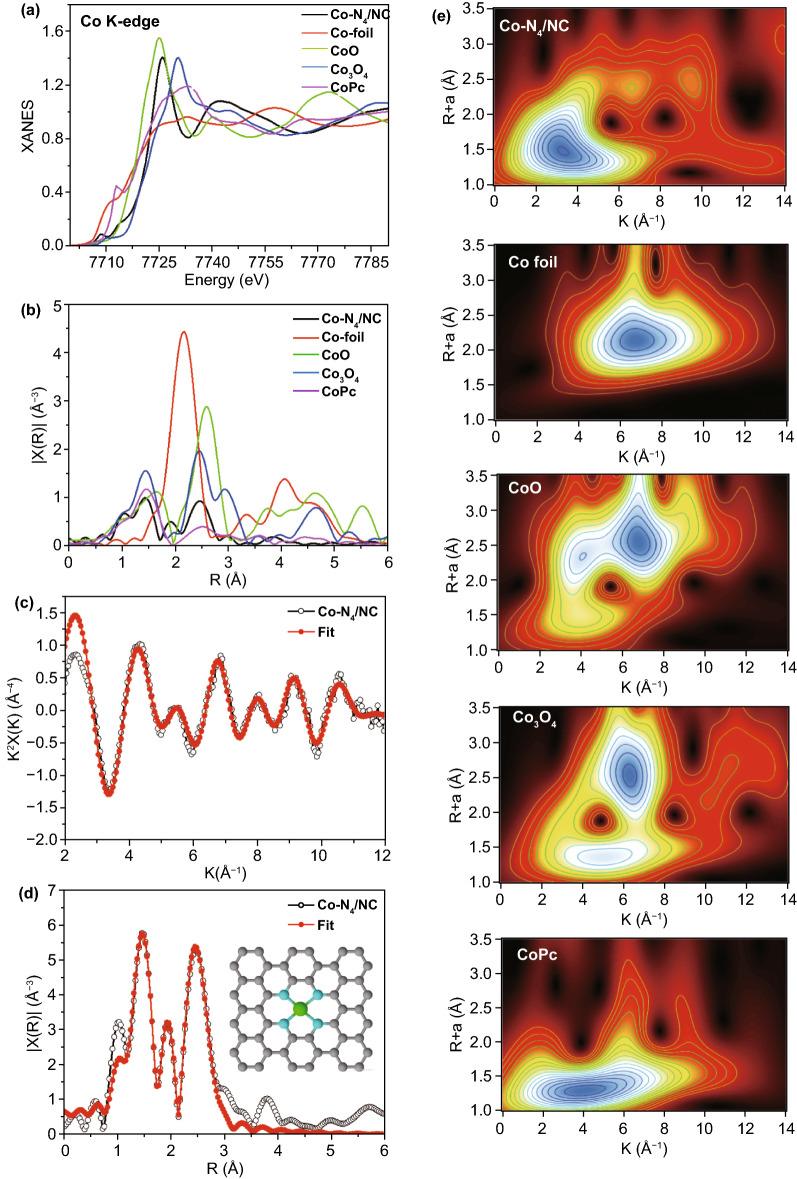
The local electronic state and chemical environment of Co-N_4_/NC. **a** Comparison of Co K-edge XANES spectra of Co foil, CoO, Co_3_O_4_, CoPc, and Co-N_4_/NC. **b** Fourier transform of the EXAFS spectra of Co foil, CoO, Co_3_O_4_, CoPc, and Co-N_4_/NC. **c**, **d** EXAFS analysis of Co-N_4_/NC at K and R space. The inset in **d** demonstrates the model derived from the EXAFS results. **e** Wavelet transform of Co K-edge XANES spectra of Co foil, CoO, Co_3_O_4_, CoPc, and Co-N_4_/NC

The metal valence states and chemical environments of M-N_4_/NCs were further probed at the atomic level by XANES and EXAFS spectroscopy. Figure 4a presents the Co K-edge XANES spectrum of Co–N_4_/NC and those of Co foil, CoO, Co_3_O_4_, and CoPc as four control references. Co–N_4_/NC presented a pre-edge peak at 7708.5 eV, a value identical to that observed for cobalt phthalocyanine (CoPc), which suggested the presence of a planar Co–N_4_ coordination environment [52, 53]. Besides, the Co peak of Co-N_4_/NC was located between those of CoO and Co_3_O_4_, i.e., the Co oxidation state lied between +2 and +3. Furthermore, the Fourier-transformed *K*^2^-weighted *x*(*k*) function of EXAFS spectra in *R*-space was used to quantify coordination numbers and bond lengths. For Co-N_4_/NC (Fig. 4b), a prominent peak at 1.47 Å was observed, corresponding to the first coordination shell of Co-N. This peak was highly similar to that of atomically dispersed Co-N bonds observed for CoPc [52, 53]. The EXAFS fitting results of Co-N_4_/NC (Fig. 4c, d) revealed the existence of Co–N bonds (peak at 1.47 Å), with the corresponding coordination number of the central Co atom determined as ~ 4, which agrees with the planar structure of Co-N_4_. (The proposed local atomic structure is illustrated in the inset of Fig. 4.) The wavelet transform was further used to analyze the Co K-edge EXAFS spectra of Co-N_4_/NC. Figure 4e shows that for Co-N_4_/NC, the maximum intensity was observed at ~ 3.5 Å^−1^, which is very close to the value of Co-N in CoPc (~ 4 Å^−1^), but quite distinct from that of Co foil (~ 6.7 Å^−1^), CoO (4 Å^−1^ < and/or < 7 Å^−1^), and Co_3_O_4_ (5 Å^−1^ < and/or < 7 Å^−1^). Therefore, Co-N_4_/NC was concluded to contain atomically dispersed planar Co-N_4_ motifs.

**Fig. 5:**
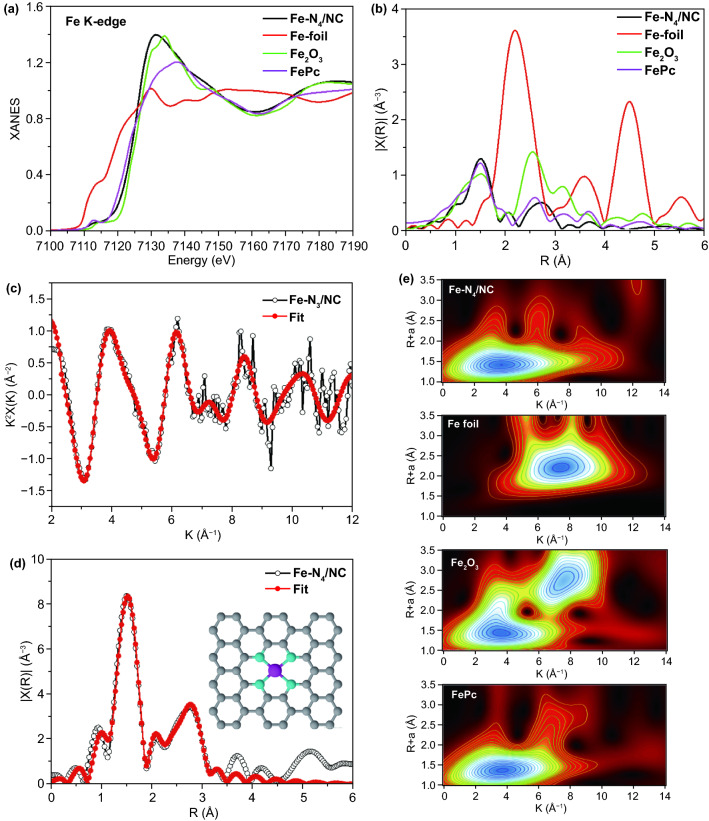
The local electronic state and chemical environment of Fe-N_4_/NC. **a** Comparison of Fe K-edge XANES spectra of Fe foil, Fe_2_O_3_, FePc, and Fe-N_4_/NC. **b** Fourier transform of the EXAFS spectra of Fe foil, Fe_2_O_3_, FePc, and Fe-N_4_/NC. **c**, **d** EXAFS analysis of Fe-N_4_/NC at K and R space, respectively. The inset in **d** demonstrates the model derived from the EXAFS results. **e** Wavelet transform of Fe K-edge XANES spectra of Fe foil, Fe_2_O_3_, FePc, and Fe-N_4_/NC

Similarly, the Fe K-edge XANES spectrum of Fe–N_4_/NC was compared to those of three reference materials: Fe foil, Fe_2_O_3_, and Fe-Pc (Fig. 5a). The spectrum of Fe–N_4_/NC featured a pre-edge peak at 7114.8 eV, which was identical to that of iron phthalocyanine (Fe-Pc) with a distinct Fe-N_4_ planar structure [54, 55]. Moreover, the peaks of Fe-N_4_/NC were located between those of Fe and Fe_2_O_3_, i.e., the above catalyst contained Fe^0^ and Fe^3+^. Figure 5b presents the Fourier-transformed *K*^2^-weighted *x*(*k*) function of the Fe-N_4_/NC EXAFS spectrum in *R*-space, revealing a prominent peak at 1.53 Å that is significantly different from that of Fe foil and Fe_2_O_3_, but is highly similar to that of planar Fe-N coordination in Fe-Pc [54, 56]. The results of Fe-N_4_/NC EXAFS spectrum fitting (Fig. 5c, d) revealed a peak of Fe-N at 1.53 Å, with the coordination number of the central Fe atom determined as ~ 4, showing that the Fe atom is coordinated to four N atoms in the planar structure. The proposed local atomic structure of Fe-N_4_/NC is presented in the inset of Fig. 5d. The wavelet transform of Fe-N_4_/NC (Fig. 5e) demonstrates only one peak with a maximum at ~ 4 Å^−1^, which is relatively close to the value of Fe-Pc (~ 3.9 Å^−1^) and completely different from those of Fe foil (~ 8 Å^−1^) and Fe_2_O_3_ (3.5 Å^−1^ < and/or < 8 Å^−1^). Therefore, Fe-N_4_/NC was concluded to contain atomically distributed planar Fe-N_4_ units. The results of detailed X-ray analyses suggest that the ultrasonication-assisted plasma engineering process allows one to preserve the unique planar Co-N_4_ and Fe-N_4_ coordination motifs within the NC matrix. Even at a low dopant content of < 0.3 wt %, the unique electronic structure and coordination environment of atomic metal-N_4_ units could apparently enhance the ORR/OER activity of carbon-based electrocatalysts.

### ORR/OER Performance

**Fig. 6:**
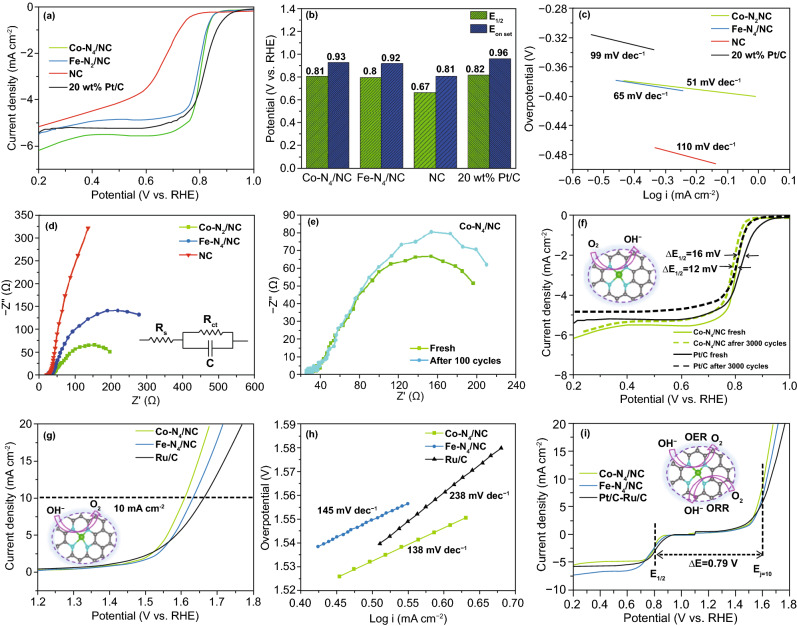
**a** Linear scan voltammetry (LSV) curves, **b** Half-wave potential and onset potential and, **c** Tafel slopes of Co-N_4_/NC, Fe-N_4_/NC, NC, and Pt/C for ORR in O_2_-saturated 0.1 M KOH solution at 1600 rpm with 5 mV s^−1^. **d** Electrochemical impedance spectroscopy (EIS) measurements of Co-N_4_/NC, Fe-N_4_/NC, and NC. **e** EIS of Co-N_4_/NC fresh and after 100 cycles. **f** ORR polarization curves of Co-N_4_/NC and Pt/C before and after 3000 cycles. **g** LSV polarization curves and **h** Tafel slopes for OER of Co-N_4_/NC, Fe-N_4_/NC, and Ru/C. **i** LSV polarization curves of different catalysts for ORR/OER in O_2_-saturated 0.1 M KOH solution at 1600 rpm with 5 mV s^−1^

The ORR and OER performances of electrocatalysts were examined using a rotating ring–disk electrode in O_2_-saturated 0.1 M KOH. The CV curves of Co-N_4_/NC and Fe-N_4_/NC featured cathodic peaks at 0.70 and 0.65 V versus RHE, respectively (Fig. S8). Figure 6a, b presents the related linear sweep voltammograms and histograms, showing that the ORR onset potential (*E*_onset_) and half-wave potential (*E*_1/2_) of Co-N_4_/NC equaled 0.93 and 0.81 V versus RHE, respectively, while the corresponding values of Fe-N_4_/NC equaled 0.92 and 0.80 V versus RHE, respectively. Both M-N_4_/NCs demonstrated significantly higher ORR activities than pristine NC (*E*_onset_ = 0.81 V and *E*_1/2_ = 0.67 V). These findings prove the importance of single-atom M-N_4_ units as efficient ORR active sites despite their very low content of < 0.3 wt %. LSV curves recorded at rotation rates of 400–2000 rpm were used to examine the ORR activity of M-N_4_/NCs (Fig. S9). Furthermore, RRDE were simultaneously collected to calculate electron transfer number (*n*) and H_2_O_2_ yield of Co-N_4_/NC, Fe-N_4_/NC and 20 wt % Pt/C. As shown in Fig. S9a, both single-atom-doped catalysts show calculated *n* values above 3.6 over the potential range of 0.2–0.8 V, revealing high ORR selectivity and efficient 4e^−^ transfer mechanism. Figure S9b illustrates that the H_2_O_2_ percentage of Co-N_4_/NC was below 29%. Figure 6c compares the ORR Tafel slopes of Fe-N_4_/NC, Co-N_4_/NC, NC, and 20 wt % Pt/C to further analyze the ORR mechanism. The Tafel slopes of Fe-N_4_/NC (65 mV dec^−1^) and Co-N_4_/NC (51 mV dec^−1^) were lower than that of 20 wt % Pt/C (99 mV dec^−1^), while NC showed a higher slope of 110 mV dec^−1^. This result indicated that the ORR kinetics of Co-N_4_/NC were superior to those of other catalysts. The electrochemical impedance spectrum of Co-N_4_/NC (Fig. 6d) presented the smallest-radius semicircle in the intermediate frequency range, i.e., this catalyst exhibited a lower charge transfer resistance than Fe-N_4_/NC and NC. Long-term cycling stability is a crucial indicator of electrocatalyst performance. Cycling did not significantly affect the electrochemical impedance spectrum of Co-N_4_/NC (Fig. 6e), although charge transfer resistance increased by ~ 10% after 100 cycles. Moreover, Co-N_4_/NC demonstrated a very weak attenuation (~ 12 mV) of *E*_1/2_ after 3000 cycles at a sweep rate of 50 mV s^−1^ (Fig. 6f). Conversely, commercial Pt/C exhibited a larger reduction of *E*_1/2_ (16 mV) after an identical durability test. These results confirm that single-atom Co-N_4_ active sites are firmly anchored within the N-doped graphitic carbon matrix with superior stability. Furthermore, the ORR stability of Co-N_4_/NC is determined by the chronoamperometry measurement at a constant potential of 0.6 V for 30,000 s (Fig. S10a) and the chronopotentiometry response at a constant current density of—4 mA cm^−2^ for 12,000 s (Fig. S10b). After the durability test, Co-N_4_/NC retains 83.6% of its initial current density in Fig. S10a, while exhibiting a slight increase in overpotential after 12,000 s at a constant current density of—4 mA cm^−2^ in Fig. S10b. Overall, the single-atom Co-N_4_/NC presents relatively high robustness and stability.

The OER activities of Co-N_4_/NC and Fe-N_4_/NC were also investigated in 0.1 M KOH electrolyte. Among all, Co-N_4_/NC features the lowest onset potential (1.52 V) and overpotential (1.60 V) at 10 mA cm^−2^, while Fe-N_4_/NC exhibited a similar overpotential of 1.63 V at 10 mA cm^−2^ (Fig. 6g). Both M-N_4_/NCs showed better intrinsic OER activity than 5 wt % Ru/C (overpotential at 10 mA cm^−2^ = 1.67 V), while NC showed the worst OER performance (Fig. S11). As shown in Fig. 6h, the Tafel slopes of Co-N_4_/NC, Fe-N_4_/NC, and Ru/C equaled 138, 145, and 238 mV dec^−1^, respectively. Thus, Co-N_4_/NC presented both the highest OER activity and the best OER kinetics. The OER stability of Co-N_4_/NC was determined by the chronoamperometry measurement at a constant potential of 1.6 V for 5000 s. Figure S12 indicates that Co-N_4_/NC shows a large attenuation after 5000 s compared to its initial current density, illustrating that there is a certain instability under the relatively high operating potential. Compared to the relatively high ORR stability Co-N_4_/NC at a potential of 0.6 V (Fig. S10), the stability at the higher potential range is much lower. The key aspects of the instability of single-atom metal catalysts are mainly due to (1) single-atom migration into cluster and (2) single-atom metal loss due to detachment [57]. Particularly, atomic metal atoms often drift on pristine carbon surfaces to form aggregate particles due to the relatively weak interactions between carbon and metal when compared to the strong *sp*^2^ binding between carbon atoms [58]. Some possible approaches have been reported, including stabilizing the single metal atoms on defective graphene [59], carbon vacancies [60], or introduction of foreign atoms such as nitrogen or sulfur into the carbon supports [61]. In particular, the atomic structure of M-N_4_ moieties was reported to anchor the single-atom metal site securely onto the carbon support [62–64]. For instance, Zhang et al. reproted a stable Co center atoms coordinated with four pyridinic N atoms of the Co-N_4_ plane [65]. Based on the XANES spectrum, ultrasonic-plasma-engineered Co-N_4_/NC catalyst presents very similar pre-edge peak to that of cobalt phthalocyanine (CoPc), which proves the existence of a planar Co-N_4_ coordination. Based on the above literature reviews, this stable Co-N_4_ structure could have avoided the migration or detachment of single-atom metal active sites from the support. In contrast, the OER stability of Co-N_4_/NC still shows low stability. Although the single-atom cobalt metal atoms are anchored strongly via the M-N_4_ carbon support, transitional metal active sites are easily oxidized during the OER process at high operating potential of 1.6 V [66]. Li et al. studied the stability of Co/Fe catalysts by in situ and *operando* X-ray absorption spectroscopy (XAS) to probe the structural and oxidation changes of transitional metal elements under 1.6 V versus RHE. Both Co and Fe showed a clear edge shift toward higher energy, which implied that the metal active sites were further oxidized compared to their original states. Furthermore, Co and Fe remain at its high oxidation state and the reaction was irreversible even the electrode potential was switched back to OCV [67].

The potential difference Δ*E* (Δ*E* = *E*_*j*=10_ − *E*_1/2_) is often used as a measure of bifunctional ORR/OER activity. Figure 6i shows that Co-N_4_/NC demonstrated the smallest Δ*E* of 0.79 V, with a slightly higher value of 0.83 V observed for Fe-N_4_/NC. For comparison, a 1:1 (w/w) mixture of commercial Pt/C and Ru/C served as the benchmark bifunctional electrocatalyst and exhibited a Δ*E* of 0.88 V, which exceeded those of Co-N_4_/NC and Fe-N_4_/NC. The intrinsic ORR/OER activities of Co-N_4_/NC and Fe-N_4_/NC seemed to be slightly inferior to those of other recently reported single-atom catalysts. Previous DFT calculations and other reports suggest that high metal loadings and well-dispersed metal atoms in M-N_4_ matrices are important for superior ORR/OER activity [68]. In contrast to other methods, ultrasonication-assisted plasma engineering allows the simple formation of a fine dispersion of isolated single-atom M-N_4_ sites. However, the main drawback of this technique is the low solubility of metal phthalocyanines in aniline and other organic solvents. Compared to previously reported single-atom-doped M-N_4_ electrocatalysts prepared by high-temperature pyrolysis of MOFs, polymers, or organic compounds, our electrocatalysts presented a small metal loading (< 0.3 wt %), but still exhibited ORR/OER activities comparable to those of isolated Fe or Co electrocatalysts with a similar mass loading (Table S3). Theoretically, the single-atom metal loading can be easily increased if other metal precursors (metal nitrides, metal hydroxides, etc.) with higher solubility in organic solvent are chosen. Also, the energy and frequency of the ultrasonic device should be designed accordingly to create more cavitation in solution media to ensure single-metal active sites can be atomically dispersed even with higher metal loading. Nevertheless, our approach is believed to be well suited for the scalable synthesis of single-atom metal electrocatalysts.

### DFT Calculations

**Fig. 7:**
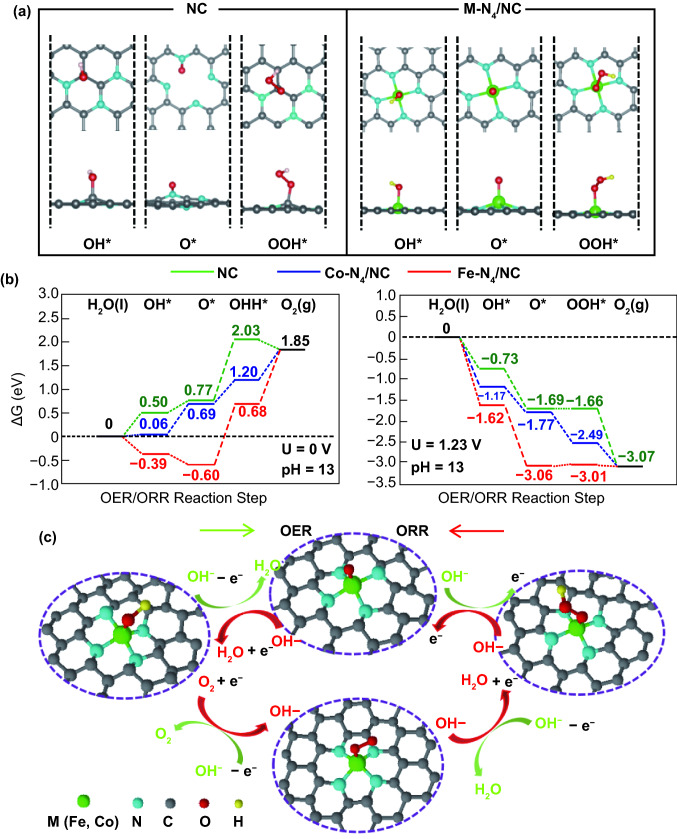
**a** Stable adsorption configuration of OER and ORR intermediates on NC and M-N_4_/NC. **b** Calculated free energy landscape for OER and ORR on NC (green), Co-N_4_ (blue) and Fe-N_4_ (red)—embedded graphene at U = 0 V (left) and 1.23 V (right). **c** Structure of OH*, O*, OOH* adsorbed M-N_4_ and the mechanisms in OER/ORR processes. The gray, blue, green, red, and yellow sphere are C, N, M (Fe, Co), O, and H atoms

DFT calculations were performed to obtain deeper insights into the free energy landscape of the ORR and OER. First, we have modeled NC structures with N-doped graphene-based supercells having low and high defect concentrations according to experimental observation of the various N-doped graphene structures. The relaxed atomic configurations of OER/ORR intermediate adsorbates on NC, Co-N_4_/NC, and Fe-N_4_/NC are illustrated in Fig. 7a. The ORR/OER reaction steps (I–IV) corresponding to OOH, O, and OH adsorption and H_2_O generation are described as follows:

*ORR reactions*

(I) O_2_(g) + H^+^ + *e*^−^ → OOH^*^
(II) OOH^*^ + H^+^ + *e*^−^ → O^*^
(III) O^*^ + H^+^ + *e*^−^ → OH^*^
(IV) OH^*^ + H^+^ + *e*^−^ → H_2_O(l) + ^*^

*OER reactions*

(I) H_2_O(l) + ^*^ → OH^*^ + H^+^ + *e*^−^
(II) OH^*^ → O^*^ + H^+^ + *e*^−^
(III) O^*^ → OOH^*^ + H^+^ + *e*^−^
(IV) OOH^*^ → O_2_(g) + H^+^ + *e*^−^

The calculated OER/ORR free energy of NC was added to compare with those of Co-N_4_/NC and Fe-N_4_/NC at U = 0 V and U = 1.23 V in Fig. 7b. In the case of NC, several reaction steps of OER and ORR were energetically uphill. For example, a positive energy is required in OER at step III (O^*^ → OOH^*^ + H^+^ + e^−^). In the case of ORR at U = 0 V and 1.23 V, the NC also exhibits a significant energy barrier at the initial step (O_2_(g) + H^+^ + e^−^ → OOH^*^). Compared to that of Co-N_4_/NC and Fe-N_4_/NC, NC shows much higher energy barriers in both OER and ORR performance, in agreement with its inferior bifunctional catalytic activity in our experimental results. As single-atom-doped M-N_4_/NCs have significantly higher OER/ORR activity than NC, one can easily assume that metal-N_4_ coordination is the dominant contributor to active sites. Previous DFT calculations also demonstrated that adsorption on single metal (Fe, Co) atoms in M-N_4_/NC supercells is much more favorable than that on N or C atoms [69]. Hence, more detailed study of the adsorptions of ORR/OER intermediates (OOH, O, OH) as well as their theoretical overpotentials on these metal sites should be considered. The theoretical overpotential of each catalysts at pH13 is calculated via the following equation:

6 $$\eta_{\text{OER}} \left( V \right) = \hbox{max} \left\{ {\Delta G_{\text{I}} ,\Delta G_{\text{II}} ,\Delta G_{\text{III}} ,\Delta G_{\text{IV}} } \right\}/e{-}0.46$$

7 $$\eta_{\text{ORR}} \left( V \right) = 0.46{-}\hbox{min} \left\{ {\Delta G_{\text{I}} ,\Delta G_{\text{II}} ,\Delta G_{\text{III}} ,\Delta G_{\text{IV}} } \right\}/e$$

where △*G*_max_ and △*G*_min_ are minimum and maximum Gibbs free energies of the four reaction step of OER.

The *η*_OER_*/η*_ORR_ of Co-N_4_ and Fe-N_4_ is calculated based on the highest energy barrier at the rate-determining step (RDS). In the case of Co-N_4_/NC, the RDS of OER is found to be the conversion of OOH* to O_2_ (OOH^*^ → O_2_(g) + H^+^ + e^−^), while the last step in ORR (OH* + H^+^ + e^−^ → H_2_O(l) + ^*^) requires the highest energy uphill. In case of Fe-N_4_/NC, the RDS of OER belongs to step III (O^*^ → OOH^*^ + H^+^ + e^−^), where step IV (OH^*^ + H^+^ + e^−^ → H_2_O(l) + ^*^) presents the greatest energy barrier in ORR. Based on their corresponding free energy, the *η*_OER_ of Co-N_4_ and Fe-N_4_ calculated from DFT calculations is 0.19 and 0.82 V, respectively. Meanwhile, the corresponding *η*_ORR_ of Co-N_4_ and Fe-N_4_ is reported as 0.40 and 0.85 V. The trends of theoretically OER and ORR overpotential from DFT calculations are well consistent with experimental results, as both overpotentials of Co-N_4_/NC obtained from electrochemical are lower than that of Fe-N_4_/NC. The theoretical and experimental overpotentials are summarized in Table S5. The detailed pathways of reversible OER/ORR reactions promoted by M-N_4_ active sites are summarized in Fig. 7c. Overall, both DFT calculations and electrochemical evaluations confirmed that single-atom Co-N_4_/NC is the most promising bifunctional OER/ORR electrocatalyst.

### Evaluation of an In-House-Built ZAB

**Fig. 8:**
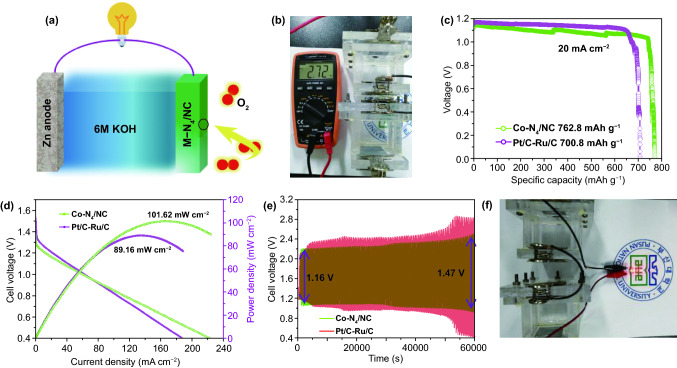
**a** Schematic of zinc–air battery. **b** Open cell voltage (OCV) of two-connected Zn–air battery. **c** Specific discharging capacities at 20 mA cm^−2^. **d** Discharging polarization and power density curves based on the Co-N_4_/NC and Pt/C-Ru/C catalyst. **e** Cycling test (100 cycles) at a current density of 10 mA cm^−2^ with Co-N_4_/NC and Pt/C-Ru/C catalyst. **f** Images of two homemade rechargeable Zn–air batteries in series with a LED

Rechargeable ZABs are promising energy storage and conversion devices, featuring the advantages of high theoretical specific capacity, intrinsic reliability, and low cost. Therefore, the performance of Co-N_4_/NC was further investigated in a practical ZAB, which comprised carbon paper loaded with Co-N_4_/NC (1 mg cm^−2^) as the air electrode and polished Zn foil as the anode (Fig. 8a). For comparison, a 1:1 (w/w) mixture of Pt/C-Ru/C was applied with the same catalyst loading. A solution containing 6.0 M KOH and 0.2 M zinc acetate was used as the electrolyte. Figure 8b shows a photograph of two in-house-made ZABs, revealing that the open-circuit voltage of Co-N_4_/NC reaches 2.72 V. Figure 8c reveals that at a current density of 20 mA cm^−2^, Co-N_4_/NC demonstrated a superior specific capacity of 762.8 mAh g^−1^ (93.0% of the theoretical capacity of 820 mAh g^−1^), which exceeded that of Pt/C-Ru/C (700.8 mAh g^−1^). The remarkable specific capacity of Co-N_4_/NC at a high current density mainly originated from the excellent conductivity and well-dispersed single-atom Co-N_4_ active sites which could significantly enhance electron transfer and ion diffusion. Figure 8d compares the discharge polarization and power density curves of Co-N_4_/NC and Pt/C-Ru/C. The discharge voltages of Co-N_4_/NC at current densities of 20, 75, and 140 mA cm^−2^ were determined to be 1.17, 0.95, and 0.71 V, respectively. At the same time, the benchmark Pt/C-Ru/C electrocatalyst exhibited discharge voltages of 1.19, 0.95, and 0.64 V at 20, 75, and 140 mA cm^−2^, respectively. Although Co-N_4_/NC showed a slightly inferior discharge voltage at lower current densities of 0–20 mA cm^−2^, its performance significantly improved and exceeded that of Pt/C-Ru/C at current densities above 80 mA cm^−2^. Besides, the maximum power density of Co-N_4_/NC (101.62 mW cm^−2^) was significantly higher than that of Pt/C-Ru/C (89.16 mW cm^−2^). These results indicate that Co-N_4_/NC is a better choice than Pt/C-Ru/C for the air electrode of practical ZABs [70]. The galvanostatic cycling performance of the fabricated ZAB was investigated at 10 mA cm^−2^ (Fig. 8e). Although the potential difference for the charge–discharge of Pt/C-Ru/C (0.95 V) was lower during the first 10 cycles, the overpotential rapidly increased to 2.5 V after 100 charge–discharge cycles (60,000 s) because of the insufficient stability of noble metal nanoparticles on the carbon matrix. In contrast, the potential difference of Co-N_4_/NC increased only from 1.16 to 1.47 V under the same condition of 100 cycles (Fig. S13). The open-circuit voltage of the Co-N_4_/NC electrode remained at 1.24 V after 100 cycles, which implies that the single-atom Co-N_4_ active sites were highly stable and stayed electrochemically active (Figs. S14 and S15). Figure 8f shows that two in-house-made ZABs with Co-N_4_/NC as the air electrode could light up a light-emitting diode when connected in series. Table S4 compares our ZAB to previously reported systems and indicates that the performance of Co-N_4_/NC was on par with that of other electrocatalysts.

## Conclusions

Ultrasonication-assisted plasma engineering was used to incorporate evenly dispersed single-atom M-N_4_ (M = Fe, Co) sites into NC, achieving a production rate of ~ 10 mg/min. The results of physicochemical analysis supported the incorporation of planar M-N_4_ structures into the NC matrix, revealing that these structures acted as the major ORR/OER active sites. In an alkaline medium, the single-atom M-N_4_/NC (M = Fe, Co) electrocatalysts showed high ORR/OER performance and stability superior to those of benchmark Pt/C and Ru/C catalysts. Specifically, Co-N_4_/NC showed a notable potential difference (Δ*E*) of 0.79 V, outperforming the benchmark Pt/C-Ru/C at the same loading (Δ*E* = 0.88 V). DFT calculations showed that the free energy landscape of Co-N_4_/NC indicated better adsorption–desorption in reversible ORR/OER stages than that of Fe-N_4_/NC, which well agreed with the experimental results. In a practical application, Co-N_4_/NC demonstrated electrocatalytic activity superior to that of Pt/C-Ru/C, achieving higher power density and durability in a rechargeable ZAB. Thus, herein we describe a promising, versatile, and scalable strategy of synthesizing various types of single-atom metal–carbon catalysts, an important class of frontier catalytic materials widely utilized in metal–air batteries and fuel cells.

## Supplementary Information

Below is the link to the electronic supplementary material.

Supplementary material 1 (PDF 796 kb)

## Abbreviations

NC: Nitrogen-doped carbon
DFT: Density functional theory
ORR: Oxygen reduction reaction
OER: Oxygen evolution reaction
ZAB: Zinc–air battery
MOF: Metal–organic framework
XRD: X-ray diffraction
FE-STEM: Field-emission scanning transmission electron microscopy
EELS: Electron energy loss spectroscopy
XPS: X-ray photoelectron spectroscopy
EXAFS: X-ray absorption fine structure
XANES: X-ray absorption near-edge structure
RHE: Reversible hydrogen electrode
LSV: Linear sweep voltammetry
CA: Chronoamperometry
CV: Cyclic voltammetry
TEM: Transmission electron microscopy
SAED: Selected area electron diffraction
HR-TEM: High-resolution transmission electron microscopy
HAADF-STEM: High-angle annular dark-field scanning transmission electron microscopy
