# Supplementary material for: Ultrasonic Plasma Engineering Toward Facile Synthesis of Single-Atom M-N4/N-Doped Carbon (M = Fe, Co) as Superior Oxygen Electrocatalyst in Rechargeable Zinc–Air Batteries
Source: Nanomicro Lett. 2021 Jan 21;13:60. doi: 10.1007/s40820-020-00581-4 (PMC8187693; doi:10.1007/s40820-020-00581-4)
Supplement: Supplementary file 1 — Supplementary material 1 (PDF 796 kb) [file 40820_2020_581_MOESM1_ESM.pdf]

Supporting Information for

Ultrasonic-Plasma Engineering toward Facile Synthesis of Single-Atom M-N_4 /N-doped carbon (M=Fe, Co) as Superior Oxygen Electrocatalyst in Rechargeable Zinc-Air Batteries

Kai Chen[1, †], Seonghee Kim[1], Minyeong Je[2, †], Heechae Choi[2, *], Zhicong Shi[3], Nikola Vladimir[4], Kwang Ho Kim[1, 5, *], Oi Lun Li[1, *]

[1]Department of Materials Science and Engineering, Pusan National University, 30 Jangjeon-dong, Geumjeong-gu, Busan 609-735, Republic of Korea

[2]Theoretical Materials & Chemistry Group, Institute of Inorganic Chemistry, University of Cologne, Greinstr. 6, Cologne 50939, Germany

[3]School of Materials and Energy, Guangdong University of Technology, Guangzhou 510006, P. R. China

[4]Faculty of Mechanical Engineering and Naval Architecture, University of Zagreb, Ivana Lucica 5, Zagreb 10002, Croatia

[5]Global Frontier R&D Center for Hybrid Interface Materials, 30 Jangjeon-dong, Geumjeong-gu, Busan 46241, Republic of Korea

†Kai Chen and Minyeong Je contributed equally

*Corresponding authors. E-mail: h.choi@uni-koeln.de (H. Choi), kwhokim@pusan.ac.kr (K. H. Kim), helenali@pusan.ac.kr (O. L. Li)

Supplementary Tables and Figures

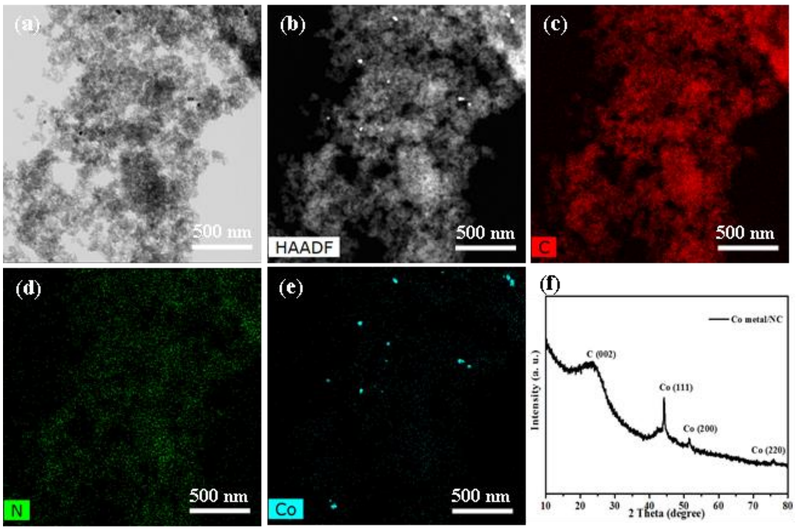

Fig. S1 a-e TEM images and corresponding HAADF mapping, and **f** XRD patterns of

conventional plasma engineering of Co-N_4 precursors in aniline without ultrasonic homogenizer

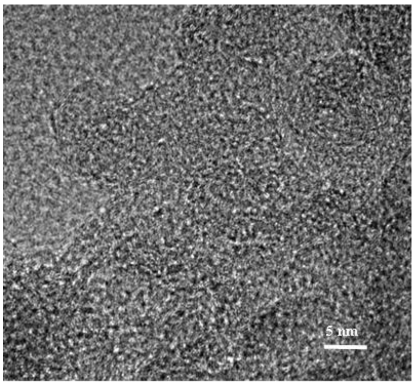

Fig. S2 HR-TEM of Fe-N_4/NC

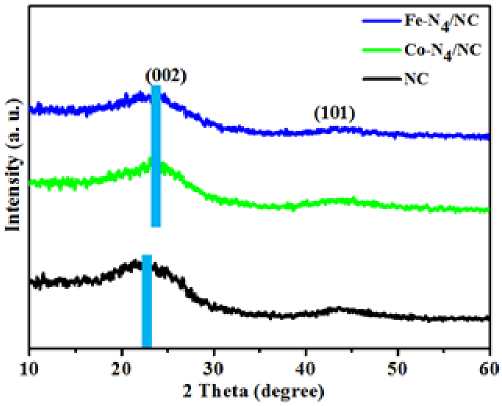

Fig. S3 XRD patterns of Co-N_4/NC, Fe-N_4/NC, and NC

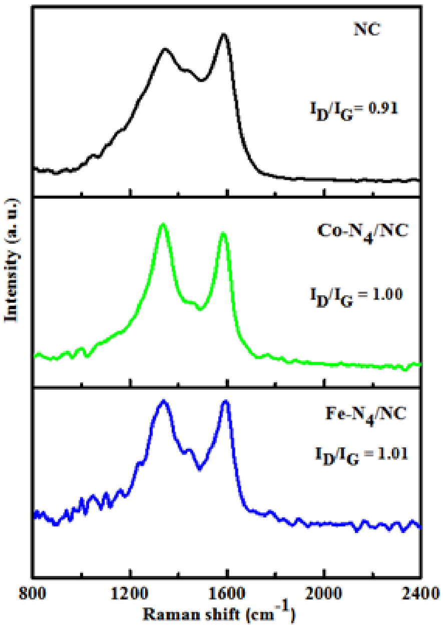

Fig. S4 Raman spectra of Co-N_4/NC, Fe-N_4/NC, and NC

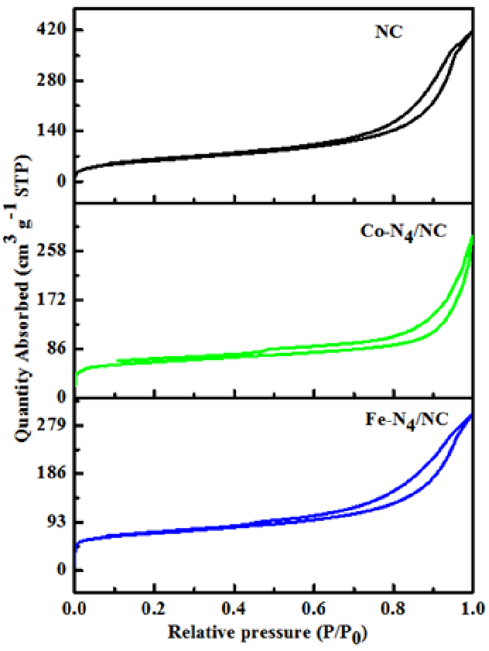

Fig. S5 N_2 adsoprtion and desoprtion curves of Co-N_4/NC, Fe-N_4/NC, and NC

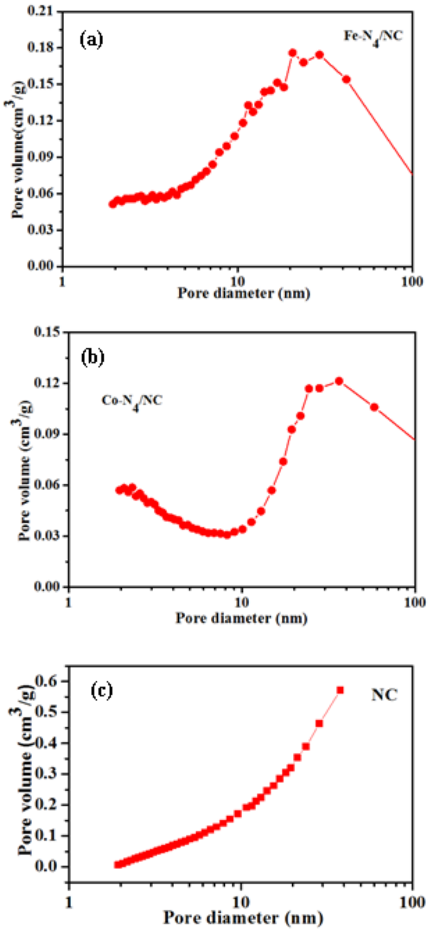

Fig. S6 Pore size distribution of (**a**) Fe-N_4/NC, (**b**) Co-N_4/NC and, (**c**) NC

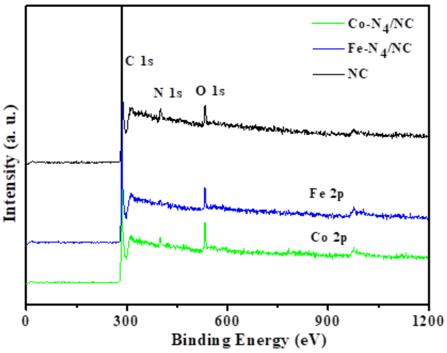

Fig. S7 XPS survey spectrum of M-N_4/NC (M = Co, Fe) and NC

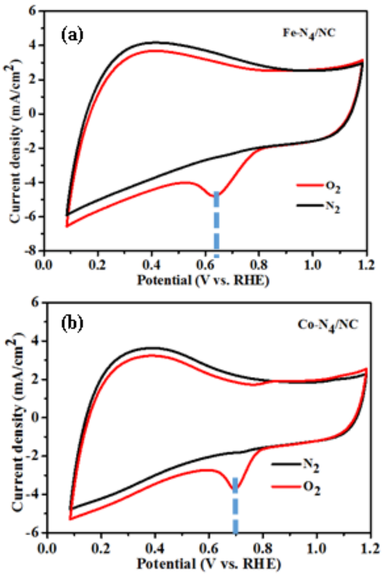

Fig. S8 CV curves recorded in O_2-saturated (red line) and N_2-saturated (black line) in 0.1 M KOH solution of Fe-N_4/NC and Co-N_4/NC

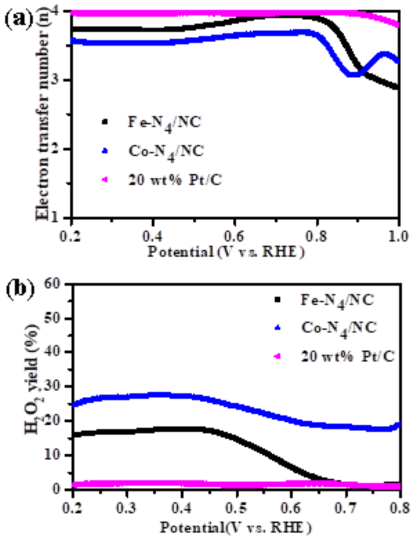

Fig. S9 a, **b** Electron transfer number and H_2O_2 yield of Co-N_4/NC, Fe-N_4/NC, and 20 wt% Pt/C

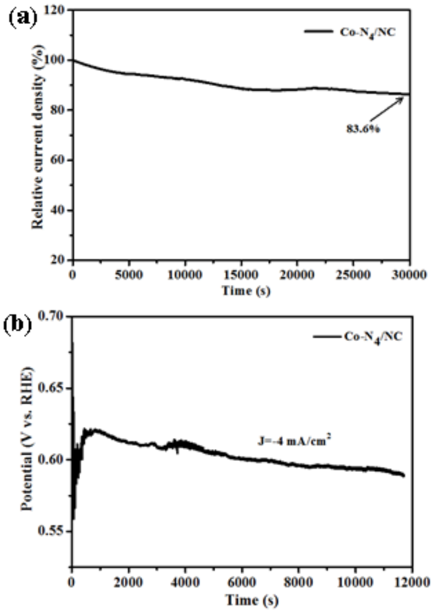

Fig. S10 a ORR chronoamperometric response of Co-N_4/NC at a constant potential of 0.6 V. **b** ORR chronopotentiometric response of Co-N_4/NC at a constant current density of -4 mA cm^{-2}

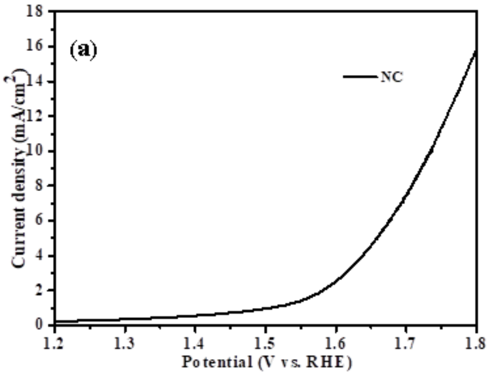

Fig. S11 OER catalytic activity (LSV) of NC at 1600 rpm in 0.1 M KOH

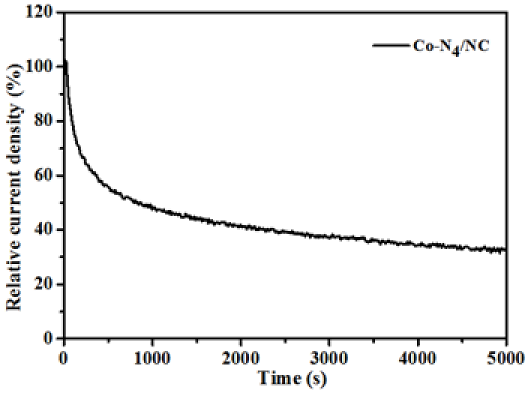

Fig. S12 OER chronoamperometric response of Co-N_4/NC at a constant potential of 1.6 V

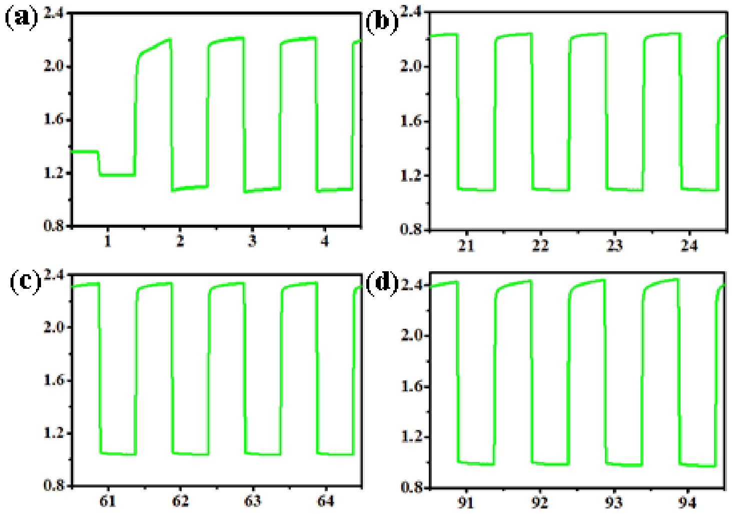

Fig. S13 a-d Discharging-charging curve of Co-N_4/NC battery in various cycle ranges

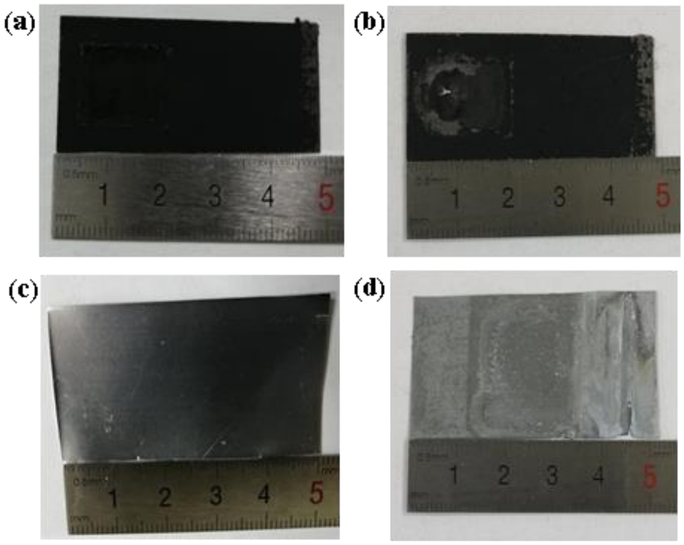

Fig. S14 a, **b** Carbon sheet and **c**, **d** zinc sheet before and after 100 cycles

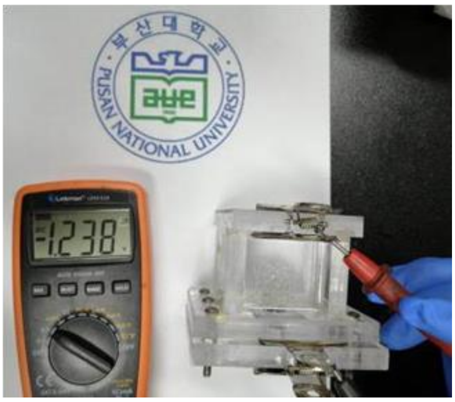

Fig. S15 OCV of used Zinc-air battery with Co-N_4/NC after 100 cycles

Table S1 XPS results of M-N_4/NC analysis for the prepared samples (at%)

Sample Name	C1s (at.%)	N1s (at.%)	O 1s (at.%)	Co2p[3] (at.%)	Fe2p[3] (at.%)
**Co-N_4/NC**	91.65	3.21	4.87	0.27	
**Fe-N_4/NC**	93.85	1.1	4.93		0.21

Table S2 Textural parameters of M-N_4/NC derived from the N_2 adsorption-desorption isotherms

Sample Name	BET surface area (m^2/g)	BJH Adsorption Pore volume (cm^3/g)	BJH Adsorption Average pore width (nm)
**Co-N_4/NC**	226.71	0.3945	14.75
**Fe-N_4/NC**	257.91	0.4261	10.95

Table S3 Summary of ORR/OER catalytic properties of reported single-atom M-N_4 catalsyts in 0.1 M KOH

Eectrocatalyst	Active sites	ORR ($E_{1/2}$) Or OER (E10mA/cm^2) vs RHE	Metal loading	References
Co-N_4/NC	Co-N_4	$E_{1/2}$ =0.81V<br>E_{10mA/cm^2} =1.60 V	< 0.3 wt.%	*This work*
Fe-N_4/NC	Fe-N_4	$E_{1/2}$ =0.80 V<br>E_{10mA/cm^2} =1.63 V	< 0.3 wt.%	*This work*
Pt/C (20 wt. %)	Pt/C	$E_{1/2}$ =0.82 V		*This work*
E-FeNC	Edge Fe-N_4	$E_{1/2}$ = 0.88 V	0.32 wt.%	[S6]
FeNC	Fe-N_4	$E_{1/2}$ = 0.84 V	0.37 wt %	[S6]
Fe-ISAs/CN	Fe-N_4	$E_{1/2}$ = 0.90 V	2.16 wt.%	[S8]
FeN_x-PNC	Fe-N_4	$E_{1/2}$ = 0.89 V<br>E_{10mA/cm^2} =1.62 V	3.935 at.%	[S10]
FeSAs/PTF-600	Fe-N_4	$E_{1/2}$ = 0.87 V	8.3 wt.%	[S5]
FeNC-S-MSUFC	Fe-N_4	$E_{1/2}$ = 0.68 -0.73 V	0.16 - 0.5 at.%	[S7]
(CM+PANI)-Fe-C	Fe-N_4	$E_{1/2}$ = 0.80 V	0.2 at %	[S9]
Fe-NCCs	Fe-N_4	$E_{1/2}$ = 0.82 V	0.26 at %	[S9]

Co-SAs@NC	Co-N_4	$E_{1/2}$ = 0.82 V;<br>$E_{10\ mA/cm^2}$ = 1.8 V	1.7 wt.%	[S2]
CoNC700	Co-N_4 planar	$E_{1/2}$ = 0.85 V	0.73 at.%	[S3]
Co-SAs/N-C	Co-N_4	$E_{1/2}$ = 0.88 V	4 wt.%	[S1]
Co-N4/NG	Co-N_4	$E_{1/2}$ = 0.87 V<br>E_{10mA/cm^2} = 1.61 V	~ 1 wt.%	[S4]

Table S4 Summary of OER and ORR overpotential of Co-N_4/NC and Fe-N_4/NC obtained from DFT and experiment

	**DFT**		**Experiment**	
	**η_{OER} (V)**	**η_{ORR} (V)**	**η_{OER} (V)**	**η_{ORR} (V)**
Co-N_4/NC	0.19	0.40	0.29	0.30
Fe-N_4/NC	0.82	0.85	0.31	0.30

Table S5 Comparison of Zn-air batteries performance of this work with recently reported similar highly active bi-functional catalytic materials [S11-S19]

**Electrocatalyst**	**Power Density (mW cm^{-2})**	**Specific capacity (mA hg^{-1})**	**OCV (V_{max})**	**Cycle life performance**	**References**
Co-N_4/NC	101.62	762.8	1.36	> 100 cycles, > 20 h	*This work*
Pt/C-Ru/C	89.16	707.9	~	~	*This work*
NiO/CoN PINWs	79.6	945	1.46	>12 h	*ACS Nano* ***11****, 2275 (2017)*
Co-Nx/C NRA	193.2	853	1.42	40 cycles, > 80 h	*Adv. Funct. Mater.,* ***28****, 1704638 (2018)*
NCNT/CoO-NiO-NiCo	~	594	1.22	100 cycles	*Angew. Chem., Int. Ed.* ***54****, 9654 (2015)*
Co@N-C	105	~	1.46	> 120 h	*Advanced Materials,* ***30*** *1705431 (2018)*
EA-Co-900	73	~	1.37	110 h	*Applied Catalysis B: Environmental*

					***256**, 117778 (2019)*
CoSx@PCN/rGO	110	634	~	394 cycles, > 43.8 h	*Adv. Energy Mater.* ***8**, 1701642 (2018)*
N-P-Fe-C	~	625	1.29	100 min	*J. Mater. Chem. A* ***4**, 8602-8609 (2016)*
NGM-Co	152	749.4	1.44	> 180 cycles, > 60 h	*Adv. Mater.* ***29**, 1703185 (2017)*

Supplementary References

[S1]P.Q. Yin, T. Yao, Y. Wu, L.R. Zheng et al., Single cobalt atoms with precise N–coordination as superior oxygen reduction reaction catalysts. Angew. Chem. Int. Ed. **55** (36), 10800–10805 (2016). https://doi.org/10.1002/ange.201604802

[S2]X.P. Han, X.F. Ling, Y. Wang, T.Y. Ma, C. Zhong et al., Generation of nanoparticle, atomic-cluster, and single–atom cobalt catalysts from zeolitic imidazole frameworks by spatial isolation and their use in zinc–air Batteries. Angew. Chem. Int. Ed. **131**(16), 5413–5418 (2019). https://doi.org/10.1002/ange.201901109

[S3]G. Wan, P.F. Yu, H.R. Chen, J.G. Wen, C.J. Sun et al., Engineering single–atom cobalt catalysts toward improved electrocatalysis. Small **15**, 1704319 (2018).

https://doi.org/10.1002/smll.201704319

[S4]L. Yang, L. Shi, D. Wang, Y.L. Lv, D.P. Cao, Single-atom cobalt electrocatalysts for foldable solid–state Zn–air battery. Nano Energy **50**, 691–698 (2018). https://doi.org/10.1016/j.nanoen.2018.06.023

[S5]J.D. Yi, R. Xu, Q. Wu, T. Zhang, K.T. Zang et al., Atomically dispersed iron–nitrogen active sites within porphyrinic triazine–based frameworks for oxygen reduction reaction in both alkaline and acidic media. ACS Energy Lett. **3**(4), 883–889 (2018). https://doi.org/10.1021/acsenergylett.8b00245

[S6]R.G. Ma, G.X. Lin, Q.J. Ju, W. Tang, G. Chen et al., Edge-sited $Fe-N_4$ atomic species improve oxygen reduction activity via boosting O_2 dissociation. Appl. Catal. B: Environ. **265**, 118593 (2020). https://doi.org/10.1016/j.apcatb.2020.118593

[S7]Y. Mun, S. Lee, K. Kim, S. Kim, S. Lee et al., Versatile strategy for tuning ORR activity of a single $Fe-N_4$ site by controlling electron–with drawing/donating properties of a carbon plane. J. Am. Chem. Soc. **141**(15), 6254 (2019). https://doi.org/10.1021/jacs.8b13543

[S8]Y.J. Chen, S.F. Ji, Y.G. Wang, J.C. Dong, W.X. Chen et al., Isolated single iron atoms anchored on N–doped porous carbon as an efficient electrocatalyst for the

oxygen reduction reaction. Angew. Chem. Int. Ed. **56**(24), 6937–6941 (2017). https://doi.org/10.1002/ange.201702473

[S9]H.T. Chung, D.A. Cullen, D. Higgins, B.T. Sneed, E.F. Holby et al., Direct atomic–level insight into the active sites of a high–performance PGM-free ORR catalyst. Science **357**(6350), 479–484 (2017). https://doi.org/10.1126/science.aan2255

[S10] L.T. Ma, S.M. Chen, Z.X. Pei, Y. Huang, G.J. Liang et al., Single–site active iron–based bifunctional oxygen catalyst for a compressible and rechargeable zinc–air battery. ACS Nano **12**(2), 1949–1958 (2018). https://doi.org/10.1021/acsnano.7b09064

[S11] C.Y. Su, H. Cheng, W. Li, Z.Q. Liu, N. Li et al., Atomic modulation of FeCo–nitrogen–carbon bifunctional oxygen electrodes for rechargeable and flexible all-solid-state zinc–air battery. Adv. Energy Mater. **7**, 1602420 (2017). https://doi.org/10.1002/aenm.201602420

[S12] J. Yin, Y.X. Li, F. Lv, Q.H. Fan, Y.Q. Zhao et al., NiO/CoN porous nanowires as efficient bifunctional catalysts for Zn–air batteries. ACS Nano **11**, 2275–2283 (2017). https://doi.org/10.1021/acsnano.7b00417

[S13] I.S. Amiinu, X.B. Liu, Z.H. Pu, W.Q. Li, Q.D. Li et al., From 3D ZIF nanocrystals to Co-Nx/C nanorod array electrocatalysts for ORR, OER, and Zn–air batteries. Adv. Funct. Mater. **28** (5), 1704638 (2018). https://doi.org/10.1002/adfm.201704638

[S14] X. Liu, M. Park, M.G. Kim, S. Gupta, G. Wu et al., Integrating NiCo alloys with their oxides as efficient bifunctional cathode catalysts for rechargeable zinc–air batteries. Angew. Chem. Int. Ed. **54**, 9654–9658 (2015). https://doi.org/10.1002/anie.201503612

[S15] M.D. Zhang, Q.B. Dai, H.G. Zheng, M.D. Chen, L.M. Dai, Novel MOF–derived Co@N–C bifunctional catalysts for highly efficient Zn–air batteries and water splitting. Adv. Mater. **30**, 1705431 (2018). https://doi.org/10.1002/adma.201705431

[S16] J. Zhao, R.X. Qin, R. Liu, Urea-bridging synthesis of nitrogen-doped carbon tube supported single metallic atoms as bifunctional oxygen electrocatalyst for zinc-air battery. Appl. Catal. B: Environ. **256**, 117778 (2019). https://doi.org/10.1016/j.apcatb.2019.117778

[S17] W.H. Niu, Z. Li, K. Marcus, L. Zhou, Y.L. Li et al., Surface–modified porous carbon nitride composites as highly efficient electrocatalyst for Zn–air batteries. Adv. Energy Mater. **8**, 1701642 (2018). https://doi.org/10.1002/aenm.201701642

[S18] W. Wan, Q. Wang, L. Zhang, H.W. Liang, P. Chen et al., N-, P- and Fe-tridoped nanoporous carbon derived from plant biomass: an excellent oxygen

reduction electrocatalyst for zinc–air batteries. J. Mater. Chem. A 4, 8602-8609 (2016). https://doi.org/10.1039/C6TA02150F

[S19] C. Tang, B. Wang, H.F. Wang, Q. Zhang, Defect engineering toward atomic Co–Nx–C in hierarchical graphene for rechargeable flexible solid Zn–air batteries. Adv. Mater. **29**, 1703185 (2017). https://doi.org/10.1002/adma.201703185
